# The theoretical role of the wind in aerosolising microplastics and nanoplastics from coastal combined sewer overflows

**DOI:** 10.1038/s41598-025-06115-5

**Published:** 2025-07-02

**Authors:** Lauren Biermann, David Moffat, Clive E. Sabel, Thomas E. Stovin

**Affiliations:** 1 School of Biological and Marine Sciences, University of Plymouth, Plymouth, PL4 8AA UK; 2 Environmental Intelligence Group, Plymouth Marine Laboratory, Plymouth, PL1 3DH UK; 3 School of Geography, Earth and Environmental Sciences, University of Plymouth, Plymouth, PL4 8AA UK; 4 Peninsula Medical School Faculty of Health, University of Plymouth, Plymouth, PL4 8AA UK

**Keywords:** Environmental sciences, Ocean sciences, Planetary science, Health care, Risk factors

## Abstract

Inhaled microplastics and nanoplastics (MNPs) have shown bio-persistence in the body, with concerning implications for human health. Airborne MNPs primarily originate from terrestrial sources, but sea air may contribute when onshore ‘aerosolising’ winds coincide with high concentrations of MNPs in surface waters. From the thousands of cities worldwide with Combined Sewer Overflows, millions to billions of MNPs can be discharged daily into rivers, estuaries, and the sea. To assess the possible links between water pollution and air quality, we analysed two years of Combined Sewer Overflows (spills) off Plymouth, UK, alongside same-day and long-term meteorological and satellite data. Winds exceeding 6.5 m/s were applied as the theoretical threshold for marine aerosol production at the sea surface. From 2022 to 2023, sewer spills into Plymouth Sound coincided with onshore aerosolising winds for a minimum of 178 days. Specifically, MNPs may have been stripped from coastal spills and blown back inland for over 1,586 hours, amounting to at least 10% of the 2-year period. Surprisingly, rainfall was too weakly correlated with spills to be a predictor, with little to no precipitation for 18% of sewer overflow events overall. In the satellite data, river plumes coincident with spills remained detectable ~ 10 km offshore, and we observed a significant degradation in winter water clarity over the past decade. Given the global footprint of outdated sewage infrastructure, our findings suggest that coastal spills—when combined with onshore aerosolising winds—may serve as an overlooked source of airborne MNPs. To better understand potential exposure pathways, it is essential that future scientific studies integrate air quality monitoring with assessments of coastal water quality.

## Introduction

The presence and persistence of microplastics ($\le$ 5 mm), small microplastics ($\le$ 20 µm), and nanoplastics ($\le$ 1 µm) in the environment is a growing problem that poses significant risk of harm to human health^1^. Much of the plastics polluting our marine environments were first used and disposed of on land^2,3^, and the oceans were previously considered a one-way sink^4,5^. Recent evidence, however, shows that when bursting bubbles and high surface winds eject or strip micro- to nanoplastics (MNPs) from surface waters into sea air, oceans can also serve as a source^6^.

A decade ago, ~ 5.25 trillion microplastics, or 93,000–236,000 metric tons, were estimated to be floating on the oceans’ surface^7^. However, substantially lower concentrations of microscopic plastic fragments and fibres were found using ship-based observations^8^. This ‘missing load’ was attributed to UV degradation, bio-degradation, ingestion, settling, and beaching; aerosolisation was not yet considered a possible removal process. The first paper to propose the theory that MNPs could be transferred from surface waters into the air was published in 2017, three years later^9^. Today, multiple lab and field studies have parameterised the transferability of different types, sizes, densities, and concentrations of MNPs^10–15^. It is now evident that, once aerosolised from the sea surface, airborne particles including microplastic fragments and fibres are highly mobile^16–23^. Over waters enriched in MNPs, an onshore gentle to moderate breeze of 5–6.5 m/s (3+ on the Beaufort Wind Scale) is sufficient for perturbing the surface, aerosolising microscopic plastics, and moving them meters to kilometres inland^7,24^. Airborne MNPs are then available to be inhaled by humans and animals situated varying distances from the marine source^25^.

Through environmental exposure, inhaled plastic fibres and fragments have shown bio-persistence in the lungs of the general population^26–28^. This has worrying implications. Aside from MNPs acting as a potential irritant in lung tissue, a range of chemicals are added to plastics during their production, and these may leach out into the body^29^. Mechanistically, MNPs also act as a ”Trojan Horse”^30^—accumulating and transporting pathogenic microbes (biofilm) and additional contaminants (corona) from the environment, including polycyclic aromatic hydrocarbons, polybrominated diphenyl ethers, pharmaceuticals, and heavy metals^29–39^. In healthy and asthmatic mice, inhalation of MNPs had detrimental impacts on the respiratory systems of both^40^. Human studies have shown the presence of inhaled fibres in nearly all malignant lung tumour tissue^41^, and significantly more microplastics were found in nasal fluids from patients with chronic rhinosinusitis than in healthy volunteers^42^. Worryingly, small microplastics and nanoplastics are generally able to move from the respiratory system into the bloodstream and to other tissues^43^. The presence of MNPs deposited from the blood into atheromatous plaques is associated with higher risk of heart attack, stroke, or death^44^. Though less is known about the impacts of nanoplastics, model, mouse, and cell line studies have shown they can cross the blood-brain barrier within hours, and prolonged exposure can lead to tissue damage and disease^39,45,46^.

Sources of airborne MNPs are likely to be predominantly terrestrial, including from roads, textiles, and dust^47^. For coastal waters to be a meaningful source of airborne plastics, buoyant MNPs first need to be present in surface waters in high concentrations. Indeed, aerosolisation levels of small microplastics have been seen to increase monotonically with concentrations present in the water^10,15,48^. In the UK, inputs of larger amounts of MNPs tend to stem from combined sewage systems that have overflowed, ostensibly in response to overwhelming rainfall. In such cases, untreated domestic and industrial effluent and runoff from gutters, drains, and roads bypasses water treatment works, and are instead flushed into rivers and the sea^49–52^. This raw or partially treated sewage and runoff is rich in MNPs^53^. Though wastewater treatment plants were not designed to separate out microplastics and nanoplastics from wastewater, overall removal success can be high, ranging from ~ 70% to 94%^54–57^. Thus, levels of MNPs in treated wastewater that is released into waterways or the sea is likely to be chronic, variable, and—though not negligible—relatively low^58^. Comparatively, combined sewage overflow events (spills) will cause an acute surge of very high levels of MNPs into riverine and coastal waters^55^. When such spills coincide with an onshore breeze capable of generating aerosols and transporting them inland^59,60^, sea air may thus become a source of breathable MNPs.

**Fig. 1:**
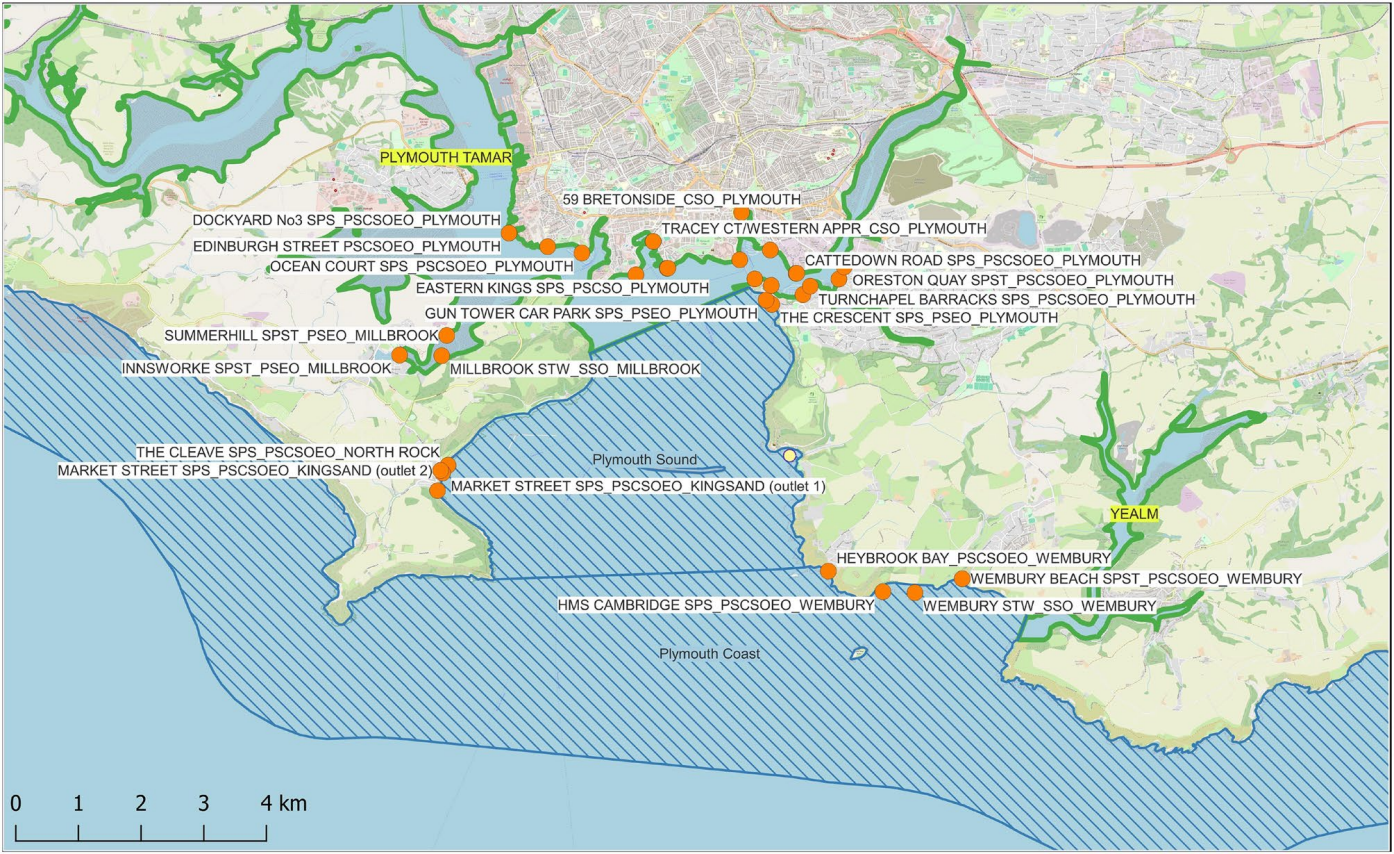
Map of the 35 South West Water (SWW) Asset sites that overflow into Plymouth Sound. SWW is responsible for the water supply and treatment of wastewater for Devon and Cornwall, and for small areas of Dorset and Somerset. EDM data were kindly provided by SWW with sites of overflows (assets) mapped by Michelle Gurney. Base map from OpenStreetMaps.

Recent analyses of global open-ocean patterns indicate that marine aerosol production, entrainment, and transport begin to increase notably at wind speeds surpassing 5 m/s^61^. Above 6.5 m/s, small breaking waves form, falling in line with thresholds for sea foam formation (indicative of substantial aerosol production) and a shift from laminar to turbulent flow (critical wind speed for air-sea boundary processes)^62–66^.

It is our primary hypothesis that, under certain conditions, sea-air may become a source of aerosolised MNPs. We consider those conditions to be met when **(1)** wastewater from Combined Sewer Overflows flushes into the sea for longer than 1 hour, and **(2)** coincident onshore wind speeds surpass a threshold of 6.5 m/s^63,64^. Using Plymouth as a case study, the primary aim of this work is to assess how often spills into coastal waters coincide with aerosolising winds blowing onshore to populated areas. Our secondary aim is to determine if rainfall can be used as a proxy for occurrence of spills (overflow events). To achieve these goals, we review two years of Combined Sewer Overflow Event Duration Monitoring (EDM) data published by the Environment Agency (EA) and shared by South West Water (SWW). Spills from the 35 overflow sites that discharge into Plymouth Sound (Fig. 1) were then assessed alongside meteorological data collected by a sea-facing National Coastwatch Institution (NCI) weather station. Supporting data included same-day winds, same-day river plume extents, and long-term water clarity derived from optical and radar data acquired by earth observation satellites.

Though this theoretical study is limited in scope to one city in the UK, its relevance is global. Combined Sewer Overflows are still used in other European countries (including the Nordics), the United States, Canada, Japan, Australia, and New Zealand.

## Methods

Standard weather parameters, including air temperature, rainfall, wind speed, and wind direction, are collected every 5 minutes from the roof of the Rame Head (RH) NCI building. These measurements are available at no charge to users as 2-day weather data archive files. We accessed all RH wind and rain data from 2022 and 2023. No weather data were collected by RH for 24 days in March 2023, and we have excluded this month from our analysis.

Sentinel-1 and Sentinel-2 are Earth Observation satellites operated by the European Space Agency (ESA) under the Copernicus Programme. The Level-1 and Level-2 optical and radar data we used in this study are freely available through a number of portals and centralised cloud-based Data and Information Access Services.

### Wind direction and wind speed

**Fig. 2:**
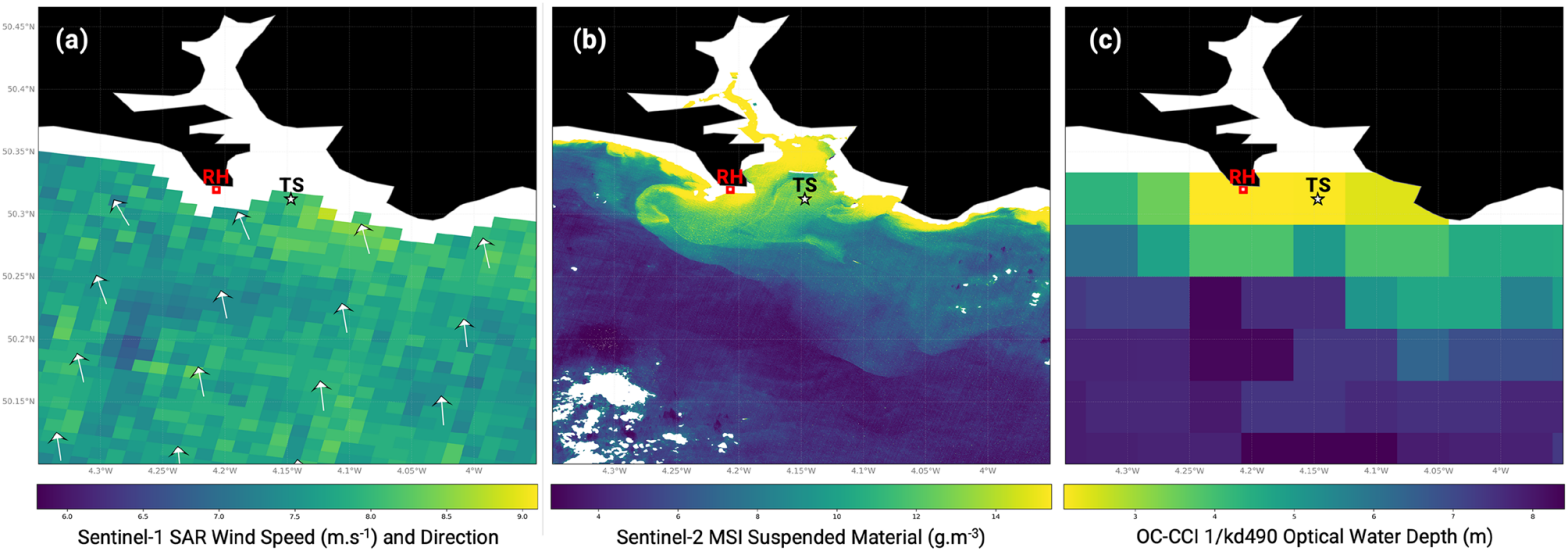
Examples of the different satellite-derived datasets used in this study, namely: SAR winds (**a**), Sentinel-2 SPM (**b**), and OC-CCI water clarity (**c**). All maps are showing data measured over November 2022. The position of Rame Head is marked on the maps as RH with a red square, and the pixel directly outside the Plymouth Breakwater is marked as TS (time-series) with a black star. The subplots in this figure were generated using Python 3.11.8.

RH is situated near a cliff edge at an elevation of 102 m. Thus, onshore wind speeds measured here will be substantially higher than those at/ near the sea surface. In order to make the two years of near continuous measurements more representative, we compared measurements from RH with those generated by satellite radar. Sentinel-1 synthetic aperture radar (SAR) Ocean Wind (OWI) is a Level-2 ground range gridded measure of ocean ‘surface’ (10 m) wind speeds and wind direction, provided at 1 $\hbox {km}^2$ spatial resolution (example shown in Fig. 2a). Sentinel-1 Level-2 Ocean files from January 2022 – December 2023 were downloaded in bulk from the Alaska Satellite Facility (ASF). A total of 177 SAR OWI data files were available over the 24 month assessment period, amounting to ~ 24% temporal coverage by the Sentinel-1A satellite.

Previous validation studies of SAR winds used *in situ* data collected at coastal stations along the Irish coastline as well as buoys at the sea surface^67^. Comparisons here showed SAR surface winds were generally underestimated by 0.4 m/s. We adjusted ‘onshore to Plymouth’ SAR winds (directions between 120 to 260 degrees) accordingly. Thereafter, a linear regression was fitted to date and time-stamped onshore SAR winds coincident *in situ* with onshore wind measurements collected at RH (WSW TO ESE). The resulting slope and intersect were applied to scale down RH winds to better represent a more ‘surface’ or near surface measurement.

### Combined sewer overflow EDM data

The EA publishes annually aggregated frequency and duration of storm overflows on the UK Government website. These Event Duration Monitoring (EDM) data are open access and available to the public. Upon request, the private utility company SWW shared timestamped (non-aggregated) EDM spill data from the 35 asset sites that empty into Plymouth Sound (Fig. 1). Before 2022, approximately 79% of SWW overflows had commissioned EDM cover. This rose to over 99% for 2022 and 100% for 2023. Thus, to reduce variability or artifacts introduced by changes in cover, we only used annually aggregated (EA) and granular (SWW) EDM data from 2022 and 2023 for our analysis.

To assess how often spills of untreated wastewater coincide with aerosolising onshore winds (co-spills), SWW EDM data were retained for analysis when RH winds blowing ESE to WSW (112.5°–247.5°) at above 6.5 m/s were measured within 12 hours of an overflow event exceeding 1 hour. Existing evidence suggests that the contents of sewer overflows, including high concentrations of buoyant MNPs, persist in surface waters for nearly a day after spill events^55^. Conservatively, we used half a day (allowing for at least one full tidal change) for our winds window. Based on these strict thresholds, and excluding March 2023, we extracted the number of co-spills per day and mean co-spill hours per month across the 35 overflow sites for 2022 and 2023. Co-spill hours were reviewed as an aggregated measure across all sites (i.e.: duration of spill measured no matter how many sites were spilling at the same time) and cumulatively (duration of sites spilling counted independently).

Finally, to ascertain if rain can be used as a predictor or ‘high flow’ proxy for future overflow events, all sewer overflow data were assessed against the 23 months of RH rainfall data using a rank biserial correlation for non-parametric data. This allowed for correlation between the binary variable (spill / no spill) and a continuous variable (amount of rainfall in the past 24 hours).

### Satellite remote sensing of plumes

The multi-spectral instruments aboard the Sentinel-2A and -2B satellites acquire optical data at a maximum of 10 m spatial resolution. Cloud-free and low glint Sentinel-2 Level-1C imagery acquired over Plymouth between January 2022 – December 2023 were downloaded from the Copernicus Data Space Ecosystem when they occurred on the same day as a co-spill event. Using ACOLITE (Atmospheric Correction for OLI ‘Lite’, 20231023.0 binary release), Sentinel-2 data were atmospherically corrected via the Dark Spectrum Fitting (DSF) approach and measures of Suspended Particulate Matter (SPM) were retrieved using the spm$\_$nechad2016 algorithm^68,69^ (example shown in Fig. 2b).

### Satellite remote sensing of water clarity

The ESA Ocean Colour Climate Change Initiative (OC-CCI) generates open access Level-3 multi-sensor composites of 1-day to monthly ocean colour products at 1 km–4 km spatial resolutions. To assess water clarity through turbidity (suspended materials) and coloured dissolved organic matter (CDOM) over time, we assessed (1) down-welling / diffuse attenuation coefficient at 490 nm and (2) the absorption coefficient for dissolved and detrital material at 412 nm (Version 6.0). 1/Kd490 is generally considered the ‘1st optical depth’^70^ where light is attenuated by approximately 63% through interaction with surface water constituents, including suspended sediment, which scatters light, and dissolved matter, which absorbs. CDOM falls into the second category, and this variable has value for tracking plumes of water rich in organic content^71,72^. With a focus on waters directly outside the Plymouth Breakwater (shown by ‘TS’ in Fig. 2), we assessed 8-day composites covering June 2014 to June 2024 (10 year period).

## Results

### Onshore winds

Over the 2-year study period, wind directions were measured as ‘onshore to Plymouth’ (120–240 degrees or ESE to WSW) ~ 45% of the time. Onshore wind speeds derived from Sentinel-1 SAR showed good linear fit with those measured from Rame Head (RH) ($\hbox {R}^2$ = 0.90) but speeds from the latter were not indicative of surface or near-surface measures due to the elevation of the station. Scaling RH measurements with estimates from SAR served to make the *in situ* wind speeds more representative, and the resulting onshore wind speeds were applied for the rest of this study.

### Co-spill patterns

Overflow events were considered coincident (hereafter designated as ”co-spills”) when a spill longer than 1 hour occurred within a 12-hour window of onshore winds above 6.5 m/s. During the 2-year period, over half of all EDM overflows met the requirement for co-spill designation.

**Fig. 3:**
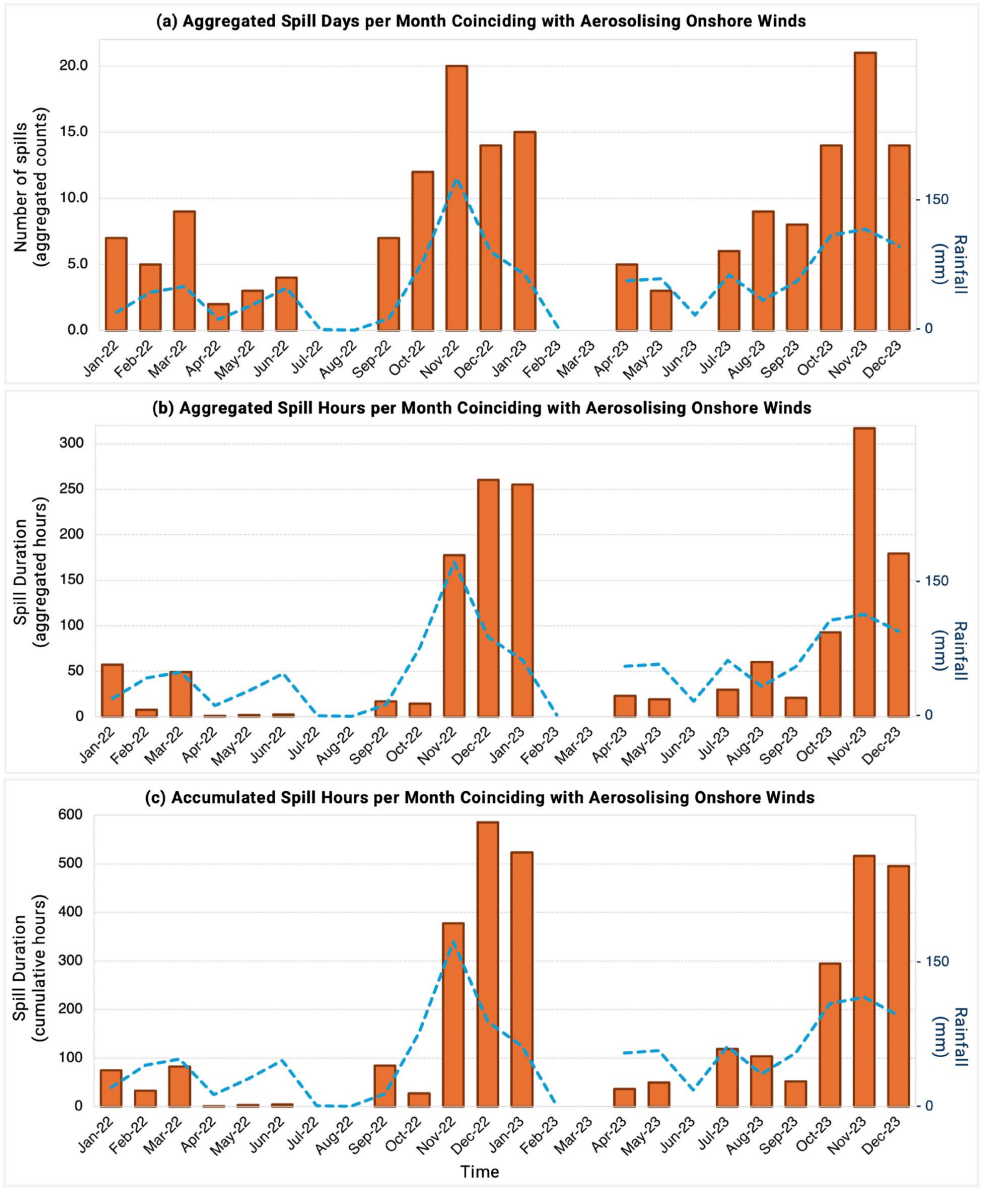
Analysis of spills that coincided with onshore winds above 6.5 m/s over the 2022 and 2023 period. Overflow events have been reported as (**a**) co-spill days per month aggregated across the 35 sites, (**b**) co-spill duration per month aggregated across the 35 sites, and (**c**) cumulative co-spill duration per month with accumulated spill hours from each site counted independently. The blue dashed line in (**a**–**c**) is illustrative of the pattern of monthly rainfall (mm) over the same period. Measures of rainfall, wind direction, and wind speed were all taken from Rame Head weather station for this analysis.

For layered insight into co-spill patterns, findings are presented in Fig. 3 as aggregated days, aggregated hours (spill hours from each site grouped together), and accumulated hours (spill hours from each site counted independently). However these spills are assessed, monthly variability is notable and a strong seasonal pattern is evident, with co-spills more recurrent and prolonged in late Autumn and early Winter months. Our findings highlight Novembers and Decembers as critical high-risk periods when water quality (and possibly air quality) is likely to be the most negatively impacted by wastewater spills. While these months also experienced higher rainfall (blue dashed line in Fig. 3), the relationship between Combined Sewer Overflows and precipitation is not straightforward.

Over the two-year period (minus March 2023), co-spills took place for an average of 89 days, 793.12 aggregated hours, or 1732.15 cumulative hours. Assuming that the majority of spills occurred due to increased precipitation, the RH measures of rainfall were assessed for their value and accuracy as a proxy for likely Combined Sewer Overflow events.

In the EA’s 2022 EDM report pertaining to SWW, over 55% of recurrent overflows were ascribed to incoming wastewater plus rainfall being at higher levels than what the sewer network could handle. Operational issues (asset maintenance) were given as the reason for 10% of recurring overflows, and 34% listed ‘N/A’ (reason unknown). Rainfall for the full year was reported as typical by the EA. In 2023, rainfall was 12% higher than average but less than 20% of overflows were ascribed to incoming wastewater plus rainfall being at higher levels than what the sewer network could handle. Approximately 14% of spills were attributed to operational issues (asset maintenance), and 55% to ‘N/A’ (reason unknown).

Our results largely support these numbers, with a significant but weak association between all spills and rainfall for the 35 asset sites ($R^{2} = 0.30$, $p < 0.001$). For 36% of spills, a maximum of 5 mm of rain had fallen within the preceding 24 hours. Little to no rain fell at all for 18% of these cases. Thus, although the relationship between rainfall and sewer overflow events is significant, it is not sufficiently strong for rainfall to serve as a reliable predictor or proxy for likely spill events—neither collectively nor on a per-site basis.

**Fig. 4:**
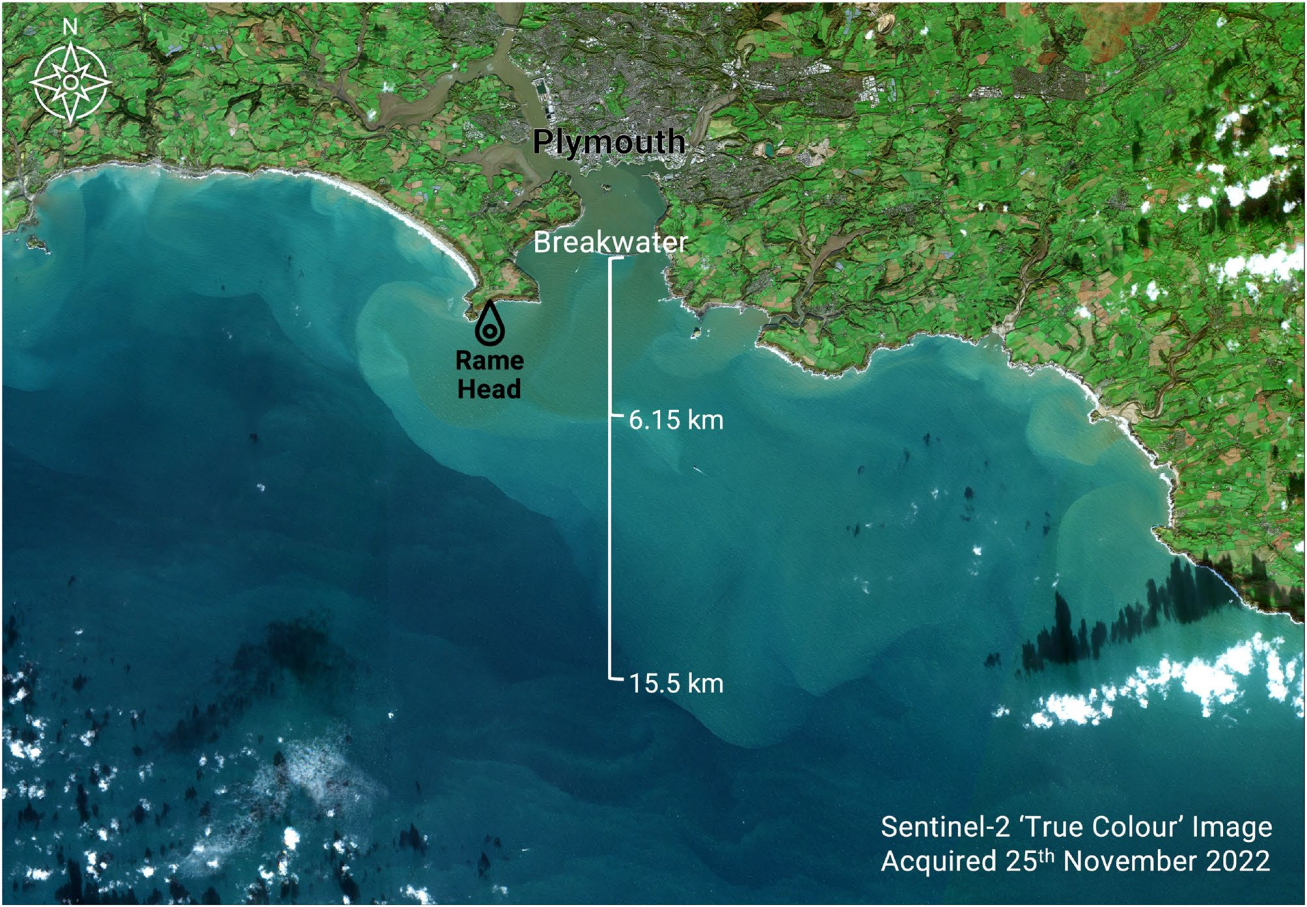
Sentinel-2 multi-spectral image acquired on the $25^{th}$ of November 2022 over Plymouth Sound. This area, including its estuaries, is designated a Special Area of Conservation (SAC). Measured from the breakwater, highly concentrated plumes of river water including spilled wastewater and other suspended / dissolved materials extend 6.15 km offshore. The more diffuse edges of the plume are still clear to the eye for another 9 km into the English Channel. Though not easily seen, whitecaps are present throughout. The overflow event/s that occurred during this time started just before midnight on the 19th and ended on the 30th of November 2022. Over this period, onshore aerosolising winds blew for over 6 hours per day for 7 of the 10 spill days. This image was generated using the Sentinel Application Platform (SNAP), version 11.

### Satellite remote sensing of plumes

Despite persistent cloud over our region of interest, high resolution optical data acquired by the Sentinel-2 satellites proved valuable for measuring how far river water mixed with untreated wastewater (and other upstream contaminants) moves offshore during overflow events (Fig. 4). This is important as winds blowing toward Plymouth appear to have extensive fetch in the SAR winds data, so they can possibly generate and entrain aerosols from surface seawater kilometres offshore before transporting them inland.

**Fig. 5:**
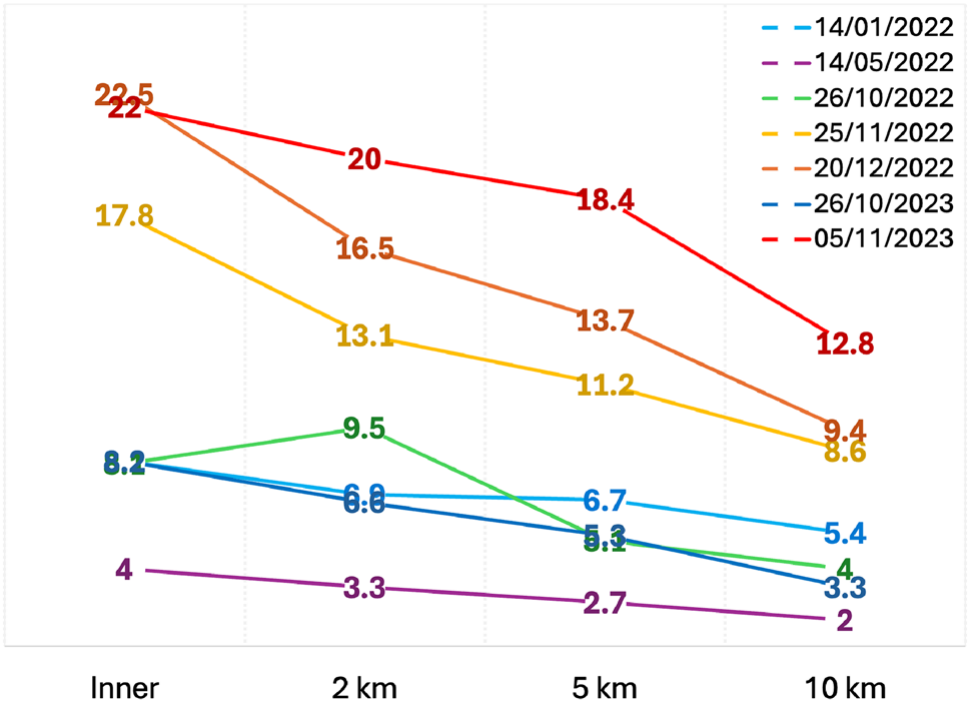
Mean suspended particulate matter (mg $\hbox {m}^{-3}$) derived from cloud-free Sentinel-2 data atmospherically corrected using ACOLITE and processed using the Nechad algorithm. Measurements were taken from inside the Plymouth Breakwater, and 2 km, 5 km, and 10 km offshore. All measurements were taken during winter, with the one exception during the summer where spills, onshore aerosolising winds, and cloud-free, glint-free Sentinel-2 cover were co-occurring (purple). This summer scene was also unique in that whitecaps were not extensive in waters close to the breakwater.

Satellite data acquisition (overpasses) co-occurred with spills 39 times over the 2-year period. Of those, only 7 scenes were sufficiently cloud and glint-free for further analysis, namely: extracting measures of suspended particulate matter (SPM). The higher co-occurrence of spills with cloudy skies was anticipated due to the association of overflows with rainfall. Winter months have the highest number of spills coincident with aerosolising onshore winds, so this time of year had more opportunities to find same-day overpasses of the Sentinel-2 satellites (Fig. 5). SPM was often highest inside and just outside the Breakwater, diluting down with distance offshore. Importantly, whitecaps were also visible within plumes. During the days with spills, onshore aerosolising winds, and useable Sentinel-2 cover, mean SPM values in plumes around 10 km offshore generally had suspended sediment concentrations at around half of those measured at the Breakwater (Fig. 5).

### Satellite remote sensing of water clarity

Directly outside the Plymouth Breakwater, 1st Optical Water Depth, as a function of water turbidity, has followed a relatively consistent seasonal pattern over the last 10 years. The clearest coastal waters are evident from May to July with a decadal average of 9–10 m. Conversely, the most turbid (unclear) waters appear to occur over the late Autumn months, with a decadal average of no more than 4–5 m. Over the last decade of Octobers and Novembers, clarity of Plymouth’s coastal waters appears to have degraded significantly ($p = 0.020$ and $p < 0.01$, respectively). Contributing to the reduced water clarity, measures of CDOM show a significant uptick over the same months ($p = 0.036$ and $p < 0.01$, respectively).

As an annual average, 1st Optical Water Depth does not show notable changes over the time-series. Interestingly, however, annually averaged CDOM does show a statistically significant increase since 2014 ($p = 0.012$), suggesting that rising concentrations of dissolved organic matter may be driving the observed reduction in Autumn water clarity over time.

## Discussion

This assessment of the role of wind as an overlooked source of aerosolised MNPs from coastal Combined Sewer Overflows is largely theoretical. What we do know with certainty is that winds above 6.5 m/s generate small breaking waves and sea foam in the open ocean^60–66^ and that MNPs, salts, trace elements, nutrients, and biological materials are aerosolised from surface waters through wind action, waves, and bubbles^7,11–15,22,23^. It is also known that untreated and partially treated wastewater are sources of extremely high concentrations of MNPs^49–58^ that can carry pathogenic microbes (biofilm) as well as environmental chemicals and contaminants (corona)^30,39,73^. Finally, a rapidly growing body of evidence demonstrates that inhaled MNPs persist in the lungs of the general population, and even ‘pristine’ MNPs without biofilms or corona have shown demonstrable negative impacts to the viability or health of cells, tissue, animals, and humans^26–28,39–46^. What is still poorly understood are the sources and exposure mechanisms.

Based on the hypothesis that sea air may be a source of MNPs during spills, we assessed how often sewer overflows off Plymouth coincided with onshore aerosolising winds. Co-spills composed more than half of all Combined Sewer Overflows in 2022 and 2023, with MNPs from discharged wastewater theoretically entering the breathable air for an average of at least 89 days each year. More specifically, at 800 hours per year, amounting to ~ 10% of the time. Establishing concentrations of airborne marine-source MNPs during such events was, however, beyond the scope of this study.

It is notable that concentrated plumes captured in high resolution optical satellite data during spills extended at least several kilometres (a few miles) offshore. This is important for two reasons. First, onshore aerosolising winds will have blown over a considerable stretch of surface waters enriched with MNPs before reaching land, and concentrations of MNPs in the air are known to increase steadily with concentrations present in the seawater^48^. Second, if Combined Sewer Overflows are contributing to the high concentrations and/or extents of these plumes, degraded water quality should be detectable in time-series data. The reduced water clarity that we measured off the Plymouth Breakwater was significant over late Autumn months, aligning with the times of year where we measured the highest duration of co-spills. Seasonal changes in turbidity of coastal waters will generally be driven by a combination of rainfall, suspended sediment, and dissolved organic matter flushed out in river water. If sewer overflows have generated higher concentrations of materials in the seawater, this may be contributing to the declining water clarity and rising concentrations of CDOM that we observed^72^. With only two years of *in situ* spill data available, it is not possible to confirm if water clarity changes are related to overflows. Nonetheless, ongoing improvements to local sewer systems and reductions in spills should result in a measurable improvement in water quality and clarity. Theoretically, these improvements would also positively impact air quality.

The second goal of this study was to assess if rainfall could be used as a proxy for likelihood of a spill, circumventing the need for sensitive data to be shared by private water companies. As per the House of Commons ‘Water Quality in Rivers’ report published on the UK Parliament website (Environmental Audit Committee, Fourth Report of Session 2021–22) Combined Sewer Overflows exist as a safety valve for use during extreme rainfall, operating to prevent sewage from backing up into homes and businesses. In the UK, spills of wastewater should, therefore, occur infrequently and under exceptional ‘high flow’ conditions^74^. Based on our assessment of spills and the amount of rainfall that had fallen in the preceding 24 hours, we have established that rain cannot be used as a predictor of spill likelihood. Though the relationship between rainfall and a spill event was significant, the association was not strong. Thus, while rainfall does contribute to overflow events in our study area, other unknown factors are also at play. This challenges the traditional assumption that Combined Sewer Overflows in the UK are primarily triggered by extreme rainfall.

With or without high rainfall, when sewage is discharged directly into coastal waters during periods of onshore aerosolising winds, sea air may become a source of breathable MNPs. Given that MNPs from seawater and wastewater act as a Trojan Horse for pathogenic organisms and/ or contaminants^9^, even a low level of exposure may pose additional risk. Though still theoretical, this presents a concerning scenario.

Outdated sewer systems have an extensive global footprint. As a known source of MNPs in water and a possible source of breathable MNPs in air, our theoretical assessment of spills may have far-reaching implications for coastal environments, and – ultimately – future human health studies. New scientific research that combines air quality monitoring with assessments of coastal water quality will be key if we are to better understand potential exposure pathways. As populations grow and climate change potentially exacerbates Combined Sewer Overflow spills, the need for proactive and integrated interventions becomes even more urgent. It is our hope that this work highlights plausible, previously overlooked links between water quality, air quality, and human exposure to airborne MNPs, informing those much-needed future *in situ* studies.

## Data Availability

The data that support the findings of this study are available from South West Water but restrictions apply to the availability of these data, which were used under license for the current study, and so are not publicly available. Data are however available from the corresponding author, L.B., upon reasonable request and with permission of South West Water.
